# The potential of video games as a pedagogical tool

**DOI:** 10.3389/fpsyg.2014.01109

**Published:** 2014-09-30

**Authors:** Brandon K. Ashinoff

**Affiliations:** School of Psychology, University of BirminghamBirmingham, UK

**Keywords:** video games, educational technology, cognitive training, plasticity and learning, games for learning, training-induced changes, training effects

When I was seven years old, my parents bought me and my brother a Nintendo Entertainment System, which they would eventually refer to as “The Idiot Box.” There was an implicit assumption (one that persists in much of the general public today) that video games were simply a toy and nothing of real substance could be gained from them. Considering this, and the prevalence of video games in society (59% of Americans play video games; Entertainment Software Association, 2014), numerous questions have been raised about the long term effects of regular use. While the media focus is generally on potential negative effects (see Ferguson and Kilburn, 2009, 2010; Anderson et al., 2010; Bushman et al., 2010; Rowell, 2010 for debate on violent video games causing aggression in gamers), there is also evidence to suggest that there may be a range of potential positive effects of video games. It should be noted that when “video games” are referenced in this article it is specifically with regard to commercial, “for fun” games and not games that were designed with educational purposes or cognitive training in mind. This article argues that video games can be a useful pedagogical tool for educators at all levels of academia.

Let's consider a simple anecdotal example of how video games may encourage children to learn large amounts of information. *Pokémon* is a popular children's game in which the player has to fight and collect various creatures, called “Pokémon.” In the original *Pokémon* game, there were 150 *Pokémon* that could be found. Each *Pokémon* has a name, a “type” (like water type or fire type), a weakness and a strength (fire types are weak against water types, but strong against grass types), and a stage of its evolution (some *Pokémon* can turn into a new one if you used it enough). Roughly speaking, this gives us 5 distinct pieces of information per *Pokémon* and multiplying this by 150 gives 750 distinct units of information contained by the list of all the *Pokémon* in the first *Pokémon* game. Now let's consider another list of information that is considered more educationally important: the periodic table of the elements. Within the periodic table each element is defined by a symbol, a name, an atomic number and weight, a phase at room temperature, and its metal or gas type. That comes to 6 distinct units of information per cell. Multiplying this by 118, the total number of elements, returns 708 distinct units of information in the periodic table. What makes this interesting is that there are a large numbers of young children (and adults for that matter) who can recite, from memory much of the information contained in this list of Pokémon, but it would likely be difficult to find the same number of people who could do that for the periodic table. The point is that the medium of video games appears to have the potential facilitate significant learning.

As it turns out, video games are designed to effectively be learning machines (Gee, 2003, 2005). If fact, many games start off with a simple tutorial level that teaches the player the basic mechanics of the game. Throughout the game, the strategy, and tactics needed to complete tasks become more complex while the teaching method gradually switches from an explicit tutorial to an experience based process. Essentially, games teach the player the skills needed to critically evaluate any situation within the game and determine the best course of action. *Starcraft 2* (as well as its predecessors *Starcraft and Starcraft: Brood War)* is what is called a “Real Time Strategy” game. Players must obtain various resources and use them to purchase buildings and fighting units. Then, they must fight and defeat their enemy. To play this game successfully, players must manage their time and resources more efficiently than their opponents as each building and unit have a different cost, purpose, and build time. Additionally, much like *Pokémon*, units have strengths and weakness. A player has to constantly update their strategies throughout the game based on their interactions with the other players, requiring significant planning and critical thinking skills that are honed by players over time. In fact, while *Starcraft* beginners make less than 100 actions (or decisions) per minute, professionals can make over 400 per minute (Lewis et al., 2011; Latham et al., 2013).

Despite evident in-game learning, one concern is that video games can only be used to teach players about game-related information and not about educationally relevant material. However, recent research into the field of digital game based learning (Prensky, 2003, 2005; Pivec, 2007; Hwang and Wu, 2012; also see Young et al., 2012) suggests that this is not the case. Squire (2005) conducted a qualitative case study on a secondary school history class in which he had students play an historical simulation game called *Civilization 3* with the intention of having the students learn about history from playing the game. It should be noted that this game was not designed specifically as an educational game (see Girard et al. (2013) for a review of the effect of intentionally educational games). In this game, students take control of a civilization (like the Aztecs or the French, for example) and progress through history developing technology, engaging in simulated warfare and diplomacy, and managing the economies of their empires. Squire (2005) reported that playing this game for educational purposes seemed to benefit students who struggled with traditional education, though students who typically performed well preferred traditional teaching methods. Here is a brief excerpt of an interview with one of the students in this study:

“Interviewer: Who do you think invented the alphabet before you played this game?

Marvin: The English, because back then they were the classiest and smartest.

Interviewer: Now who do you think invented the alphabet?

Marvin: Probably the Egyptians with the hieroglyphics. It was the first writing to be done.” (Squire, 2005)

When you play *Civilization 3*, you have to develop technology based on a set path. For example, you are required to invent the alphabet before you invent other things, like formal mathematics. Therefore, it is possible for this simple video game mechanic to teach players that the alphabet had to be invented in the first place and that it was invented before other things. Therefore, developing science in the game gives students a better idea of when things occurred in history.

Another way in which video games are learning machines (Gee, 2003, 2005) is that they are highly motivating and therefore they can induce higher student engagement compared to traditional teaching methods. According to Hamlen (2013), “despite assumptions that children play video games to avoid mental stimulation, children are actually motivated by the challenge and thinking required by video games.” Squire (2005) reported that Marvin voluntarily, and without provocation, spent time learning more about history from the “civelopedia” which provides players with historically accurate information about their chosen civilizations and other aspects of the game. Improved student engagement using video games was also demonstrated by Stansbury and Munro (2013; Stansbury et al., 2014) who supplemented an undergraduate-level behavioral statistics lecture by having students play the game *Dance Dance Revolution (DDR)* to generate scores that would be used as dependent variables while teaching the students about factorial research designs. They found, based on a pre-test/post-test comparison, that students who played *DDR* as part of their lecture showed a greater increase in content knowledge compared to students who received a traditional lecture on the same topic. Even through the inherent content of *DDR* was not educationally relevant, clever pedagogical use of the game had the desired effect of increasing student engagement with the lecture content.

## Learning to learn

There are also potential positive effects of video games that could have a less direct influence on learning and education. Harlow (1949; also see Green and Bavelier, 2008) coined the term “learning to learn,” which refers to “the process of developing skills that facilitate learning in other contexts…” (Bisoglio et al., 2014). Critically, there is evidence that video game training can influence numerous skills and abilities that are crucial to the learning process. For example, Kühn et al. (2014) trained 48 non-video game players (*M*_*Age*_ = 24.1; *S*_*Age*_ = 3.8) on Super Mario 64 for 30 min a day for 2 months. Super Mario 64 is a game in which the player must explore the game world, fight monsters, solve puzzles, and collect stars to progress. Gray matter volume was measured pre- and post- training and it was found that the volume of gray matter in the right dorsolateral pre-frontal cortex was significantly increased post-training. There is evidence to suggest that an increase in cortical volume due to video game training is related to improvements in the concomitant cognitive functions of that brain region (Basak et al., 2011; Voss et al., 2012). The dorsolateral pre-frontal cortex is has been frequently implicated in executive functions—including working memory (Goldman-Rakic, 1995; Bechara et al., 1998; Levy and Goldman-Rakic, 2000; Petrides, 2000; Curtis and D'Esposito, 2003), inhibitory control (Knight et al., 1999; MacDonald et al., 2000; Ridderinkhof et al., 2004), and attentional shifting (Nagahama et al., 2001; Kondo et al., 2004)—all of which are arguably critical to the learning process in an educational setting. There is also a wealth of behavioral evidence that video game training influences cognitive abilities including executive function (Maclin et al., 2011; Mathewson et al., 2012; Strobach et al., 2012; Anguera et al., 2013), spatial attention (Green and Bavelier, 2003, 2006a,b, 2007; Feng et al., 2007; Dye et al., 2009; Hubert-Wallander et al., 2011), selective attention (Wu et al., 2012; Belchior et al., 2013; Wu and Spence, 2013), distractor processing (Mishra et al., 2011; Krishnan et al., 2013), and attentional capture (West et al., 2008; Chisholm et al., 2010). There is even evidence to suggest that video gamers generate more robust internal representations of visual information (Green and Bavelier, 2004; Karle and James, 2011; Sungur and Boduroglu, 2012). However, a caveat is that due to inconsistent methodologies across the research in the field there is still debate over whether or not there is a causal relationship between playing video games and improved cognition (Boot et al., 2008, 2011, 2013; Boot and Simons, 2012; Schubert and Strobach, 2012; Kristjánsson, 2013; Latham et al., 2013; Bisoglio et al., 2014). That being said, overall the evidence appears to be trending toward there being a causal relationship.

In addition to the cognitive skills necessary for learning, appropriate social skills are necessary for success in education because education in general is a highly social experience. It should be noted that although cognitive and social skills are being separated in this article for the sake of clarity, they are deeply interrelated abilities in practice. Being a student (or a teacher for that matter) requires constant interaction and communication with other people. This interaction can take the form of a lecture, group assignments, study groups, or even general emotional and social support. An often overlooked aspect of video games is that they too can be a highly social experience. Despite this, a common misconception about video gamers is that they shun and avoid social engagement (Jenkins, 2005). Consider that many modern video games are multiplayer online games. For example, World of Warcraft is what is known as a massively multiplayer online role playing game (MMORPG). In this game, to defeat enemies and to progress through the game, players must interact and coordinate with each other. In some cases the execution of a sophisticated strategy, involving up to 40 players, is required to win a battle. It has been suggested that MMORPG's can provide a medium through which one can learn and practice social skills (Ducheneaut and Moore, 2004, 2005; Yee, 2006; Zhong, 2011). Jang and Ryu (2011) analyzed survey data from 300 Korean MMORPG players (*M*_*Age*_ = 25.4; *S*_*Age*_ = 5.9) about their gaming habits and their leadership experiences (both online and offline). The results revealed a positive correlational relationship between online leadership experiences and with offline leadership experiences (each measured using a different leadership questionnaire). While correlations only allow for a limited interpretation there is converging evidence to suggest that the video game play may be a causal or at least influencing factor. Yee (2003), based on a survey analysis of 2804 MMORPG players (age not reported), reported that almost half of all subjects subjectively felt that they had improved their leadership skills, defined by four subcategories, at least a little bit as a result of their gaming experiences: Mediation (55.2%), Motivation (48.4%), Persuasion (43.8%), and Leadership (50.3%). Interestingly, being in a leadership or management role in real life did not seem to affect the rate at which subjects reported improved leadership skills, suggesting playing MMORPGs could have social benefits for a wide range of people.

Additionally, competitive online team games also provide an excellent medium for enhancing social skills, particularly teamwork, and collaboration. A game mentioned earlier, *Starcraft 2*, has a highly in depth competitive culture and multiplayer community (as do games like *League of Legends* and *DOTA 2*). Poling (2013) conducted a study in which he taught an entire course using *Starcraft 2* as the primary mode of instruction throughout. According to Poling (2013) “the StarCraft 2 course encouraged learners to create new knowledge by synthesizing what they know and learn in the game world with how they can apply those skills and concepts to their real-life professional world. It used StarCraft 2 as a digital sandbox where they had to learn to work well with others in order to succeed.” Based on interviews from three participants, it was expressed that they felt they had enhanced their knowledge regarding “collaboration, teamwork, and leadership” as a result of the course. Poling (2013) reported that “one of the most important lessons they learned from both the in-person and online collaboration processes was that collaboration is essentially about managing human relationships, maintaining effective communication, and learning how to work with others despite their differences.”

Video games have also been shown to be able to influence pro-social behaviors. Greitemeyer and Osswald (2010) conducted a study in which they had subjects (*M*_*Age*_ = 21.81; *S*_*Age*_ = not reported; university students) play one of four games. In the interest of brevity, I will focus on only two. One of those games was called Lemmings and was chosen because they researchers deemed it a pro-social game. In this game, Lemmings drop from an entrance and walk forward unless there is something in their path, in which case they turn around. If the player does nothing, they will walk off the edge of cliffs and die. The purpose of the game is to guide the lemmings safely to the exits using a limited number of skills. It was deemed pro-social because the focus of the game is helping the lemming survive. The game Tetris was used as a neutral-social game. In this game, players must arrange geometrical shapes into horizontal lines to earn points. Players were asked to play their respective game for 8 min. After playing, they were presented with various social situations and their responses were recorded. The subjects were unaware that these were part of the study. In one study, the experimenter knocked some pencils onto the floor while talking to the subject. In another (which used a different pro-social game), a male confederate entered the room and verbally and physically harassed a female experimenter as if he were an ex-boyfriend who couldn't accept that their relationship was over. In both cases, the subjects who played the pro-social game were more likely to help the experimenter than the subjects who played the neutral game. The point is video games can have a positive influence on social behaviors (Durkin and Barber, 2002; Lenhart et al., 2008; Dalisay et al., in press), at least when played in moderation (Przybylski, 2014). Prosocial video games like lemmings can foster altruism, which could be good for encouraging students to help each other if one of them is struggling in class, and multiplayer games like *World of Warcraft* or *Starcraft 2* can foster the social skills needed to coordinate and cooperate with other people, which could enhance student's ability to engage in group assignments.

Video games have significant potential as a pedagogical tool. In order to begin to explore this potential, the common preconceptions about what video games are and their value must be re-evaluated. In general, there are two rules of thumb that one should keep in mind when considering how to use video games in an educational context. First and foremost, it matters what games you are playing. Puzzle games like *Portal*, may improve problem solving skills, but do nothing to improve social skills or attentional processing. Multiplayer team games like *League of Legends* may improve the ability to communicate and cooperate with groups, but may not affect interest in learning more about school work. Historical simulation games, like the *Civilization* series might enhance a student's motivation to learn more about history, but have no influence over executive functions. It is therefore important to pick and choose the games you may want to use carefully. And second, video games cannot replace a teacher or a curriculum, but judicious use of the appropriate games can complement an educational program.

### Conflict of interest statement

The author is a scientific consultant for a company that specializes in cognitive training video games.
